# Advances in spinal cord imaging in multiple sclerosis

**DOI:** 10.1177/1756286419840593

**Published:** 2019-04-22

**Authors:** Marcello Moccia, Serena Ruggieri, Antonio Ianniello, Ahmed Toosy, Carlo Pozzilli, Olga Ciccarelli

**Affiliations:** Queen Square MS Centre, NMR Research Unit, Department of Neuroinflammation, UCL Queen Square Institute of Neurology, Faculty of Brain Sciences, University College London, London, UK; Multiple Sclerosis Clinical Care and Research Centre, Department of Neurosciences, Federico II University of Naples, via Sergio Pansini, 5, Edificio 17 - piano terra, Napoli, 80131 Naples, Italy; Department of Human Neuroscience, Sapienza University of Rome, Italy; Department of Human Neuroscience, Sapienza University of Rome, Italy; Queen Square MS Centre, NMR Research Unit, Department of Neuroinflammation, UCL Queen Square Institute of Neurology, Faculty of Brain Sciences, University College London, London, UK; Department of Human Neuroscience, Sapienza University of Rome, Italy; Queen Square MS Centre, NMR Research Unit, Department of Neuroinflammation, UCL Queen Square Institute of Neurology, Faculty of Brain Sciences, University College London, London, UK; National Institute for Health Research, University College London Hospitals Biomedical Research Centre, London, UK

**Keywords:** advanced, atrophy, lesions, MRI, multiple sclerosis, spinal cord, quantitative

## Abstract

The spinal cord is frequently affected in multiple sclerosis (MS), causing motor, sensory and autonomic dysfunction. A number of pathological abnormalities, including demyelination and neuroaxonal loss, occur in the MS spinal cord and are studied *in vivo* with magnetic resonance imaging (MRI). The aim of this review is to summarise and discuss recent advances in spinal cord MRI. Advances in conventional spinal cord MRI include improved identification of MS lesions, recommended spinal cord MRI protocols, enhanced recognition of MRI lesion characteristics that allow MS to be distinguished from other myelopathies, evidence for the role of spinal cord lesions in predicting prognosis and monitoring disease course, and novel post-processing methods to obtain lesion probability maps. The rate of spinal cord atrophy is greater than that of brain atrophy (−1.78% *versus* −0.5% per year), and reflects neuroaxonal loss in an eloquent site of the central nervous system, suggesting that it can become an important outcome measure in clinical trials, especially in progressive MS. Recent developments allow the calculation of spinal cord atrophy from brain volumetric scans and evaluation of its progression over time with registration-based techniques. Fully automated analysis methods, including segmentation of grey matter and intramedullary lesions, will facilitate the use of spinal cord atrophy in trial designs and observational studies. Advances in quantitative imaging techniques to evaluate neuroaxonal integrity, myelin content, metabolic changes, and functional connectivity, have provided new insights into the mechanisms of damage in MS. Future directions of research and the possible impact of 7T scanners on spinal cord imaging will be discussed.

## Introduction

Multiple sclerosis (MS) is a chronic inflammatory demyelinating disease of the central nervous system (CNS).^1^ Spinal cord abnormalities are common in MS, and include a variety of pathological processes, such as demyelination, neuroaxonal loss and gliosis, ultimately resulting in chronic motor, sensory and autonomic dysfunction.^1,2^

Recent improvements in magnetic resonance imaging (MRI) acquisition protocols and post-processing have overcome some of the limitations associated with imaging such a small and mobile structure, whose imaging is affected by motion artefacts, caused by breathing, cardiac movement, cerebrospinal fluid (CSF) pulsation and blood flow.^3^ Conventional spinal cord MRI provides information on focal lesions, which is necessary for the diagnosis and prognosis of MS, and is commonly used in the clinical setting. Advanced quantitative MRI techniques assess the type and extent of spinal cord abnormalities, but their use is essentially limited to research centres.

The aim of this review is to present and discuss advances in spinal cord imaging in MS. We have divided the review into the following sections: (1) Spinal cord lesions on conventional MRI for MS diagnosis, prognosis and clinical monitoring, and advances in spinal cord imaging to improve visualization of lesions; (2) Spinal cord atrophy in relation to MS clinical features, and advances in imaging analysis methods for spinal cord atrophy measurement; and (3) Quantitative imaging techniques to obtain insights into the pathogenesis of MS. Finally, we will consider novel developments in spinal cord MRI, including the advent of 7T scanners, and suggest future areas of research.

## Spinal cord lesions

Spinal cord lesions on MRI correspond to areas of demyelination, neuroaxonal loss and gliosis, affecting spinal cord structure and function.^4,5^ Postmortem spinal cord studies have described a larger proportion of demyelination in the grey matter (33%) than in the white matter (20%), with lesions involving either both grey matter and white matter, or grey matter isolately.^6^ No difference in the extent of grey matter demyelination was seen between different cord levels.^6^

### Characteristics of MS lesions on spinal cord MRI

**Figure 1.**
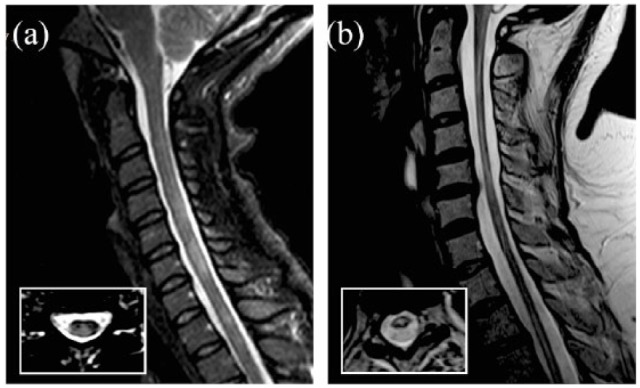
Lesions in MS and NMO. T2 sagittal and axial (inset) spinal cord MRI of a patient with MS and a patient with AQP4-antibody-positive NMOSD. In MS (a), MRI shows areas of T2 hyperintensity which extend for a single vertebral level, involve both grey and white matter in the lateral-posterior part of the cord and have a cylindric shape on the sagittal view and a wedge shape on the axial view. In AQP4 NMOSD (b), there is a longitudinally extensive transverse myelitis from C1 to C5 and a lesion at T2–T3, with preferential involvement of the central spine. MRI, magnetic resonance imaging; MS, multiple sclerosis; NMO, neuromyelitis optica; NMOSD, neuromyelitis optica spectrum disorder.

Spinal cord lesions are visualized as areas of T2 hyperintensity (Figure 1) and, less commonly, as areas of T1 hypointensity on conventional spin-echo sequences. Although T1 hypointensity in the spinal cord is thought to be rare in MS, a recent study using inversion-recovery prepared fast field echo sequence (e.g. heavily T1-weighted sequence) at 3T demonstrated that 87% of patients with MS show T1 hypointense lesions in the spinal cord, and most of the lesions seen on the short-tau inversion-recovery T2-weighted sequence were hypointense on T1.^7^

After administration of gadolinium, new inflammatory activity, with associated blood–brain barrier breakdown, allows the MS lesions to appear as areas of T1 hyperintensity; gadolinium enhancement in the acute spinal cord lesions is generally nodular and, in 20% of the cases, may have a ring shape.^8–10^

MS lesions often occur in the cervical region (59%), and, less frequently, in the lower thoracic spinal cord (T7–12; 20%).^11^ On sagittal scans, they appear as cylindrical lesions, while on axial images they appear as wedge-shape lesions. In sagittal views, they rarely exceed two vertebral segments in length. On axial scans, MS lesions involve less than 50% of the cross-sectional area, occupy preferentially the lateral and posterior white matter columns and do not spare the grey matter. However, spinal cord involvement can be diffuse, as shown by diffuse signal abnormalities on proton density (PD) images, especially in the progressive forms of MS; diffuse signal abnormalities in relapsing–remitting MS (RRMS) are associated with a poor prognosis.^12,13^

### Recommended spinal cord MRI protocols

The protocols recommended for spinal cord MRI in the clinical setting include both sagittal and axial scans.^14^ For sagittal imaging, conventional or fast dual-echo spin-echo sequences (PD and T2-weighted, either in combination or independently) are usually considered the gold standard. A recent study at 1.5T suggested that PD fast spin-echo sequences detect cord lesions in patients who have a normal T2 fast spin-echo MRI, and should therefore be used as a core sequence at 1.5T.^15^ Either the T2 or PD spin-echo sequence can be substituted with a short-tau inversion-recovery (STIR) T2-weighted sequence, which improves the visibility of MS lesions.^16^ In general, it is not recommended to use the STIR sequence on its own because of its susceptibility to flow-related artefacts and possible lower observer concordance.^17^ An alternative to the STIR sequence or to one of the two dual-echo T2-weighted sequences, is a heavily T1-weighted sequence,^18^ such as phase-sensitive inversion-recovery (PSIR) or magnetization-prepared rapid gradient echo (MPRAGE), which improves the detection of MS lesions in the cervical cord.^19^ The three-dimensional (3D) acquisition of the MPRAGE permits multiplanar reconstruction that facilitates the delineation of lesions.^20^ A recent 3T study has reported that a 3D double inversion-recovery (DIR) sequence for the cervical spinal cord imaging is more sensitive at detecting inflammatory lesions than conventional two-dimensional (2D) T2-weighted turbo spin echo (TSE) sequence.^21^ However, the DIR sequence of the spinal cord is not widely used in clinical practice because it is strongly affected by artefacts, especially in obese patients, and by magnetic field inhomogeneities, and its coverage capability is currently limited to the cervical spine.^21^

For axial imaging, possible sequences are 2D or 3D T2-weighted fast spin-echo sequences. A full cervical cord axial coverage detects more lesions (9–22%) than sagittal scans alone,^11,22^ and can also exclude lesions in cases of equivocal abnormalities on sagittal scans.^11,22,23^

No significant improvement in lesion detection was found when using 3T field strength compared with 1.5T.^24^ Improvements in lesion detection are expected at 7T, although its application and relevance requires further studies, especially in the context of new coil designs and optimized acquisition times.^2,25,26^

Although pathological involvement of the spinal cord grey matter contributes significantly to disability in RRMS and secondary progressive MS (SPMS),^27^ its assessment with conventional MRI techniques is not achievable because of insufficient contrast between tissue compartments and low spatial resolution. In the research setting, improved delineation of cervical cord lesions and their involvement of the white and grey matter are obtained by using 3D-PSIR in combination with axial 3D gradient-echo fast field echo (3D-FFE),^28^ although this MRI protocol requires a long acquisition time and has limited coverage of the cervical cord.

### Diagnosis of MS supported by spinal cord MRI

The 2017 revised McDonald criteria confirmed that MRI is the most useful paraclinical test to aid the diagnosis of MS, and can be used to establish dissemination of lesions in space (DIS) and time (DIT) in patients presenting with a clinically isolated syndrome (CIS).^29^ The spinal cord is one of the four areas of the CNS where lesions with characteristics typical of MS are scored to confirm DIS. Prior to the 2017 McDonald criteria, only asymptomatic spinal cord lesions were scored for DIS, which led to the high specificity of the DIS criteria; in order to facilitate the scoring of the criteria, and avoid discussing which lesion is the symptomatic one in cases of multiple lesions occurring in the same CNS location, the 2017 revised criteria no longer distinguish between symptomatic and asymptomatic lesions when testing the DIS criteria.^29^ The inclusion of spinal cord symptomatic lesions for DIS or DIT increases diagnostic sensitivity, with little or no reduction in specificity.^30–32^

While brain MRI is recommended in all patients who are undergoing investigations for the diagnosis of MS, spinal cord MRI is advised when: (1) The clinical presentation suggests a spinal cord lesion; (2) The clinical presentation is suggestive of primary progressive MS (PPMS); (3) Brain MRI is normal, but there is a strong clinical suspicion of MS; (4) Brain MRI findings are inconclusive (e.g. ageing).^18,29,33^ Therefore, spinal cord MRI is generally recommended in patients with spinal cord CIS and in those with nonspinal MS not fulfilling the DIS criteria. It is debated whether all the remaining CIS patients, who have nonspinal MS and fulfil DIS criteria on brain MRI brain, should undergo spinal cord MRI.^34^

More recently, patients with clinical features typical of MS, but showing evidence of pathology exclusively in the spinal cord, even with a single lesion, and whose MRI does not fulfil the DIS criteria, have been described as two novel clinical entities: (1) Progressive solitary sclerosis, when insidiously progressive upper motor neuron impairment can be attributed to an isolated demyelinating lesion within the CNS (within the spinal cord in 90% cases)^35^; and (2) Pure spinal MS, when relapsing episodes of short-segment myelitis occur over time, in the absence of typical brain or optic nerve lesions.^36^ Progressive solitary sclerosis and pure spinal MS are proposed novel MS phenotypes, characterized by a predominant spinal cord pathology.

### Differential diagnosis of myelopathies facilitated by spinal cord MRI

MS could be responsible for up to 50% cases of inflammatory myelopathies and, thus, a number of conditions should be considered in the differential diagnosis. These include neuromyelitis optica spectrum disorders (NMOSDs), myelin oligodendrocyte glycoprotein-antibody (MOG-Ab)-associated disease, sarcoidosis, and paraneoplastic syndrome, and require different treatment and management strategies.^37,38^ Certain lesion characteristics on spinal cord MRI may aid the clinicians to navigate through the differential diagnosis of spinal cord inflammatory pathology.^14^

NMOSD is responsible for up 50% cases of longitudinally extensive transverse myelitis (LETM), defined as T2 hyperintense spinal cord lesions extending ⩾3 vertebral levels (Figure 1).^38^ How-ever, the length of spinal cord lesions in NMOSD depends on the timing of MRI with respect to clinical onset.^39^ NMOSD can also present with the involvement of <3 vertebral segments.^40^ Additionally, LETM is not a pathognomonic feature of NMOSD, and other inflammatory demyelinating conditions can cause a LETM.

One of the most important spinal cord MRI features differentiating NMOSD from MS, and other LETM aetiologies, is the presence of bright spotty lesions (BSLs),^41,42^ defined as lesions with signal intensities at least as high as, but not higher than, that of the surrounding CSF on a T2-weighted image, and not as low as that of the surrounding CSF on a T1-weighted image. BSLs are seen in the majority of patients without LETM, and it is thought that they indicate severe damage to the spinal cord. Other spinal cord distinctive features of NMOSD are lesions occupying ⩾50% axial cross-sectional area (transversally-extensive lesion), T1 hypointense lesions, and centrally located or both centrally and peripherally located lesions.^38^ Gadolinium enhancement is common in NMOSD, but variable in its appearances (frequently irregular and punctuate); ring enhancement is seen in one-third of NMOSD myelitis episodes and distinguishes NMOSD from other causes of longitudinally extensive myelopathies, but not from MS.^10^ Additionally, NMOSD lesions are more frequently located in the cervical or dorsal spinal cord, when compared with the lumbar cord.^38^

Overall, 20–40% of NMOSD patients negative for the aquaporin-4 antibody (AQP4-Ab), are instead MOG-Ab positive.^43,44^ The LETM of MOG-Ab-associated disease is virtually indistinguishable from that of NMOSD AQP4-positive disease.

Spinal cord sarcoidosis is an under-recognized cause of LETM and can precede symptoms of systemic and pulmonary sarcoidosis. Linear dorsal subpial enhancement extending ⩾2 vertebral segments and persisting >2 months differentiates spinal cord sarcoidosis from NMOSD and MS, where gadolinium enhancement is patchy, diffuse or ring-like.^45,46^ When linear dorsal subpial enhancement is combined with central canal enhancement in cases of sarcoidosis, a ‘trident’ sign on axial post-gadolinium sequences has been described.^46,47^

Other important causes of spinal cord lesions are post-infectious myelitis [e.g. cytomegalovirus (CMV), herpes simplex virus, varicella zoster, enterovirus], which often present with LETM, and are associated with variable radiological appearances on T2, T1 and post-contrast T1-weighted images.

Noninflammatory myelopathies include vascular aetiologies (e.g. acute spinal cord infarction), spinal dural arteriovenous fistula, tumours, nutritional deficiencies, infections, and compressive myelopathies. In these cases, timely diagnosis and management is crucial to improve clinical outcomes.^37,48^

Additional clinical (e.g. hyper-acute or gradually progressive onset), radiological (e.g. presence of lesions on brain MRI, abnormalities on chest positron emission tomography imaging), laboratory (e.g. presence of AQP4 and MOG-Abs) features might be necessary to establish the exact diagnosis of myelopathy.^38,45,47^ The most striking consequence of a more appropriate and widespread use of spinal cord MRI and additional tests in cases of spinal cord myelopathy is that the recognition of an ‘idiopathic’ transverse myelitis is reducing over time.^37^

### Prognosis of MRI using spinal cord MRI

In patients with radiologically isolated syndrome (RIS), the presence of asymptomatic spinal cord lesions is seen in 64% of patients who later develop CIS or MS, and in 100% of patients who later develop PPMS.^49^

In patients with CIS, the presence and the number of spinal cord lesions are associated with increased risk of clinical conversion to MS and disability progression, regardless of demographics, clinical features and brain MRI.^50–52^ In contrast, the probability of disability progression is very low in the absence of spinal cord lesions.^50^

In established MS, spinal cord lesions are associated with a higher risk of relapse,^53^ disability progression,^54,55^ and switching of disease modifying treatment due to poor treatment response.^56^ Also, upper cervical cord lesion load, quantified on PSIR sequences, is greater in progressive forms of MS than in RRMS, and is associated with disability.^54^ In SPMS, spinal cord lesions frequently involve at least two spinal cord white matter columns and extend to the grey matter.^13^ The main limitation of these studies is that spinal cord coverage was confined to the upper cervical cord, in order to minimise physiologic artefacts and enable high-resolution acquisitions within an acceptable time frame, thus limiting generalizability to clinical practice.^13^

### Monitoring MS with spinal cord MRI

Spinal cord lesions are more likely to be symptomatic and leave residual neurological impairment, due to poor compensatory capacity of the spinal cord, than brain lesions.^52,53^ Despite this, 58% of new spinal cord lesions were reported to be asymptomatic and 25% of patients with RRMS develop at least one asymptomatic spinal cord lesion over 1.5 years.^57^ When only patients with stable RRMS are considered, 10% of them show subclinical spinal cord lesion activity alone.^53^ Interestingly, asymptomatic spinal cord lesions predict clinical relapses when combined with asymptomatic brain lesions.^53^ Thus, spinal cord MRI could disclose subclinical disease activity in otherwise clinically stable MS, and could enhance a more thorough understanding of the course of MS.^58^ Asymptomatic spinal cord lesions may not be restricted to patients with MS, as they have also been observed in patients with NMOSD,^59^ but more data for NMOSD are needed.

### Spinal cord lesion mapping

**Figure 2.**
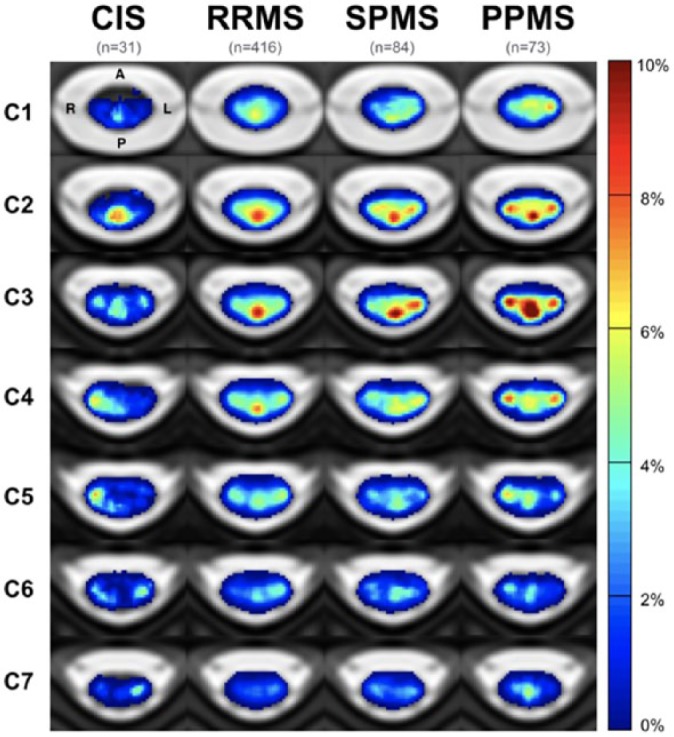
Lesion probability maps in the spinal cord. Lesion probability maps at the cervical level are shown for different disease subtypes (from Eden and colleagues^61^). CIS, clinically isolated syndrome; PPMS, primary progressive multiple sclerosis; RRMS, relapsing–remitting multiple sclerosis; SPMS, secondary progressive multiple sclerosis.

Recent developments in imaging post-processing have allowed the creation of probability maps of spinal cord lesions, which show the probability of each voxel being ‘lesional’. Single-centre studies combining 3D T1-weighted FFE and the active surface model (ASM), a semi-automated voxel-based analysis of the spinal cord, showed that patients with SPMS and especially PPMS have higher lesion counts and volumes, when compared with RRMS, and that lesions are more frequently located in the posterior cord than in the anterior cord, and in the upper cervical cord than in the lower cord.^7,60^ A larger, multicentre study, employing fully automated methods based on the Spinal Cord Toolbox (SCT) confirmed that lesions are more frequently located in the posterior columns in all MS subtypes, and that lesion mapping at C3 clearly distinguishes between MS subtypes.^61^ In particular, high lesion probability was found in the posterior columns in RRMS, posterior and lateral cord in SPMS and posterior, lateral and central regions in PPMS (Figure 2).^61^ Interestingly, high disability levels were associated with lateral and central cord involvement.^61^

## Spinal cord atrophy

An increasing number of studies have focused on the importance of spinal cord atrophy as a biomarker of disability progression and as an outcome measure in clinical trials.

### Pathology correlates

Spinal cord atrophy is the consequence of different pathological processes, including axonal transection and associated neuroaxonal loss, demyelination, gliosis, and, ultimately diffuse tissue injury.^62–64^ Although these pathological abnormalities occur within focal lesions, extensive tissue abnormalities are also present in the normal-appearing spinal cord of MS patients, and this finding may explain why spinal cord atrophy occurs independently of spinal cord lesions.^5,63–65^ Additionally, spinal cord atrophy also occurs, at least in part, independently of brain pathology.^2,66,67^

### Advances in spinal cord atrophy measurements

Spinal cord atrophy is generally measured as the cross-sectional area at the cervical level, which is least affected by movement artefacts, yields the most reproducible results, and provides the best clinical correlates.^68–72^ The most common levels are C1–C2 and C2–C3, but measurements can be also made between C1 and C7.^73^

Atrophy assessment can be done on a variety of sequences, mainly 3D T1-weighted and T2*-weighted gradient-echo sequences on different MRI scanners (e.g. Philips, Siemens, GE).^3,74,75^

Methods for spinal cord image segmentation and atrophy calculation can be classified into three types: intensity-based, surface-based and image-based.^76^ The older methods were fully manual, while the most recent methods have been semi-automated or fully automated. For example, JIM is a surface-based method that automatically outlines the cord, after marking the centre of the spinal cord.^77^ Within the JIM tool, the ASM has provided more prompt and reproducible measures of the spinal cord volume, compared with manual methods.^78^ The ASM offers a considerable reduction in user interaction time, and can be performed over long spinal tracts. The user needs to identify landmarks at the extremes of the region to study, and, then, mark the centerline of the cord. Sagittally acquired images are then reformatted to the axial plane to obtain five contiguous 3-mm slices; the program automatically calculates the radius and the centre of each axial slice and, finally, the cross-sectional area is obtained by averaging these contiguous slices.^74^ Other semi-automated method is NeuroQLab (an image-based method that segments the upper cervical cord from surrounding nonspinal cord tissue by using a Gaussian mixture modelling method).^79,80^

Recent efforts have attempted to develop fully automated methods, such as the SCT, which is an open-source comprehensive software dedicated to the processing of spinal cord MRI. SCT is built on previously validated methods and includes motion correction tools, templates and algorithms to segment the spinal cord, allowing standardization and automation of the processing pipeline.^81^ The segmentation tool (PropSeg) contained in the first version of SCT has already been tested on a large cohort of MS patients and healthy controls. This fully automated intensity-based image segmentation method has the same sensitivity as the ASM but has higher inter-rater reproducibility and is more time efficient.^82^ A newer version of SCT also includes a fully automated framework for intramedullary lesion segmentation, presenting with higher efficiency and reproducibility in lesion count and volume, when compared with manual detection.^83^

A recent study has demonstrated that there is a systematic difference in the values of the cross-sectional area between methods, with lower values provided by fully automated methods (SCT) than semi-automated methods (NeuroQLab and JIM)^84^; a good agreement between these two semi-automated techniques was observed.^84^

When using these methods for spinal cord atrophy calculation, the rate of atrophy is estimated by numerical subtraction of spinal cord cross-sectional area measurements calculated at different time points. We have recently applied to the spinal cord a registration-based technique, called the generalized boundary shift integral, used for computing brain atrophy, and have demonstrated that this method is feasible and may produce a reduction in sample size needed in clinical trials.^85,86^ It is expected that the development and optimization of registration-based techniques for spinal cord atrophy will reduce measurement noise, as it has happened when registration-based techniques were first used for computing brain atrophy.^87^

There has been a recent shift towards calculating spinal cord atrophy using brain volumetric images.^80,88^ A recent MAGNIMS study has confirmed that the spinal cord cross-sectional area, calculated at the C1–C2 level using dedicated volumetric MRI of the spinal cord, is similar to that obtained using volumetric brain MRI.^84^ Further studies will aim to validate this new approach, which has the potential to allow calculation of spinal cord atrophy without acquiring a dedicated cord sequence, thereby saving scanning time, in both clinical trials and observational cohorts.

### Spinal cord atrophy in disease phenotypes

**Figure 3.**
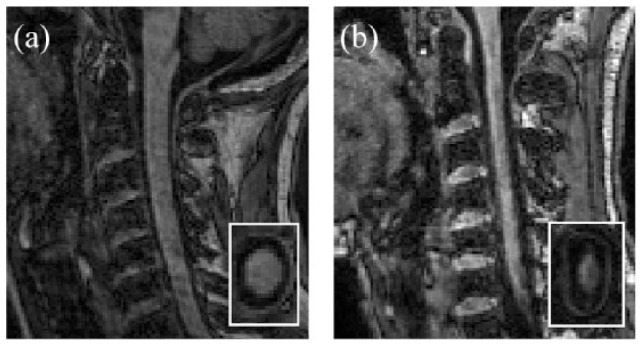
Spinal cord atrophy visible on conventional MRI. Cervical cord MRI with sagittal and C2 axial (inset, used for spinal cord cross-sectional area measurements) views in CIS (a) and PPMS (b). CIS, clinically isolated syndrome; MRI, magnetic resonance imaging; PPMS, primary progressive multiple sclerosis.

Spinal cord atrophy occurs even in early stages of MS, and has been detected in patients with CIS.^75,89–91^ In CIS patients who were followed up for 5 years after onset, the lowest rate of spinal cord atrophy (−0.1% a year) was observed in those who remained with a CIS, while the highest rate (−1.4% a year) was detected in patients who developed MS and had an expanded disability status scale (EDSS) at the last time point equal or greater than 3.^52^ In general, a high rate of spinal cord atrophy is observed in the progressive forms of MS, especially SPMS (−2.2% per year; Figure 3).^73,91^ A recent study has reported yearly rate of spinal cord atrophy between −0.38% in RRMS and −0.62% in SPMS.^92^ A MAGNIMS multicentre study has detected a rate of −1.22% per year in patients with stable MS and −2.01% in patients who deteriorated over time.^73^ Interestingly, we found a significant development of spinal cord atrophy in early PPMS patients when compared with healthy controls over only 1 year of follow up, but not in patients with established SPMS, who had a higher disability and more atrophic cord than early PPMS patients.^93^ Although the rate of atrophy may vary slightly between studies, because of different cohorts and different methods, it is consistently reported to be higher than the rate of brain atrophy, which is known to be around −0.5% per year in MS patients.^94^ A recent meta-analysis of 22 longitudinal studies assessing spinal cord atrophy in all MS subtypes revealed a pooled rate of spinal cord atrophy of −1.78% per year, that increased to −2.08% per year when considering progressive patients alone.^68^

The segmentation of grey matter areas on PSIR images at 3T allows the evaluation of grey matter atrophy in MS. Relapsing MS patients show smaller spinal cord grey matter areas (i.e. higher atrophy) than age and sex-matched controls, without significant differences in spinal cord white matter areas^95^; the grey matter of progressive MS patients shows the highest degree of atrophy.^95^

Only a few studies have examined cervical cord atrophy in NMOSD and reported conflicting results. Some studies found more pronounced spinal cord atrophy in AQP4-positive patients than patients with MOG-Abs,^96^ and in MS than NMOSD,^97^ whereas another study found similar reductions of cross-sectional areas in NMOSD and MS.^98^

### Spinal cord atrophy and MS disability

A number of studies have shown associations between: (1) the extent of spinal cord atrophy at a single time point and concurrent disability,^99^ and (2) the rate of spinal cord atrophy over time and disability progression.^52,75,91,100–102^ A recent study has reported that every 1% increase in the annual rate of spinal cord volume loss is associated with a 28% risk of developing disability progression in the subsequent year.^92^ In a longitudinal cohort of nonspinal CIS, upper cord cross-sectional area decrease was associated with 5-year increased disability, measured by the EDSS.^52^ Within the EDSS, the subscores that reflect the neurological functions mediated by spinal cord pathways, such as the pyramidal, sensory, bowel and bladder functional scores, correlated with spinal cord atrophy.^103^ A higher spinal cord atrophy rate is associated with a worsening of more specific measures of motor disability, such as the nine hole peg test (9HPT) and the 25-foot walking test (25FWT).^92,99^ Associations between the development of spinal cord atrophy and disability progression are particularly strong in PPMS.^104^

### Spinal cord atrophy in clinical trials

Since spinal cord atrophy rates are two-to-three-times higher than brain atrophy (−1.78% *versus* −0.5% per year), in particular in progressive MS,^68,105^ and the spinal cord is a very eloquent site of pathology in MS, spinal cord atrophy has been considered as an exploratory outcome measure in phase II and phase III clinical trials, especially in patients with progressive MS, although much less frequently than brain atrophy.^106^ However, clinical therapeutic trials that incorporated spinal cord atrophy as an outcome measure did not demonstrate beneficial drug effects on this metric.^107–110^ In addition to the possibility that the medications tested were not effective, there may be other reasons for these negative results, related to methodological difficulties of calculating spinal cord atrophy; these include: movement artefacts and subsequent image noise; the limited spatial resolution of MRI scanners, which is an important issue, given the small cord size; multicentre design, with inter-site variability related to the use of different scanners with different acquisition settings; and inter-study variability related to the use of different methods to calculate spinal cord area.^111,112^ Also, spinal cord normalization using the intracranial volume, which aims to reduce the effect of biological conditions unrelated to the disease, has been suggested,^69,70,113^ but it is not always performed.

There have been encouraging results from recent, single-centre, phase II clinical trials employing spinal cord atrophy.^93,111^ We demonstrated that if patients at the early stage of PPMS, with mild disability and a nonatrophic cord are selected, the sample size necessary to run a trial over only 1 year is achievable.^93^

## Quantitative spinal cord imaging techniques

Advanced imaging techniques are currently used in exploratory studies to investigate microstructural abnormalities which reflect neurodegeneration, and to develop new targets for therapeutic intervention.^114,115^ These techniques include methods that study neuroaxonal integrity [diffusion tensor imaging (DTI) and new models of diffusion], myelin content [magnetization transfer ratio (MTR), and myelin water imaging], metabolic changes [magnetic resonance spectroscopy (MRS)], and functional connectivity [functional MRI (fMRI); Table 1]. However, advanced MRI techniques remain technically challenging, and results from studies using different acquisition protocols are difficult to compare.^116–118^ We will focus on the most recent advances in these techniques and their latest applications to MS patients, and refer to other manuscripts for more technical and comprehensive reviews.^119–121^

**Table 1.** Pathological specificity of advanced spinal cord MRI and clinical correlates. Table shows pathophysiologic mechanism of MS that can be studied with different advanced MRI techniques. Changes occurring in different MS subtypes and clinical correlates are presented.

**Pathophysiologic mechanisms**	**Advanced MRI technique**	**Changes in MS** (compared with controls)	**Clinical correlates** (if abnormal)	**Reference**
**Neuroaxonal integrity**	**DTI** *(FA, RD, MD)*	MD, FA = in RIS RD ↑↑↑ in RRMS and SPMS MD ↑↑↑ in RRMS and SPMS FA ↓↓↓ in RRMS and SPMS	EDSS Upper limb function Lower limb function	^27,116,117^
	**QSI**	ADCxy, FWHMxy, P0xy ↑↑↑ in PPMS ADCz, FWHMz, P0z ↓↓↓ in PPMS	Spasticity Postural instability Sensory dysfunction	^122,123^
	**NODDI** *(v_in_, ODI)*	v_in_ ↓↓↓ in RRMS lesions ODI ↑↑↑ in RRMS NAWM ODI ↑↑↑ in RRMS lesions		^124,125^
	**SMT** *(V_ax_)*	V_ax_ ↓↓↓ in RRMS		^126^
	**MRS** *(NAA/Cr)*	↓↓↓ in PPMS, RRMS and SPMS	EDSS Upper limb function Lower limb function	^127^
	**MRS** *(NAA)*	↓↓↓ in PPMS	EDSS Spasticity Postural instability Sensory dysfunction	^122^
	**MRS** *(Glx)*	↓↓↓ in PPMS	Postural instability	^122^
**Myelin content**	**MTR**	↓ in RIS ↓↓↓ in RRMS	EDSS	^116,128,129^
	**Myelin water fraction**	↓↓↓ in PPMS ↓↓↓ in RRMS	EDSS Lower limb function	^130,131^
	**MTSat**	n/a	EDSS Lower limb function	^132^
**Astrocytic activation and proliferation**	**MRS** *(Myo-inositol)*	↑↑↑ in PPMS lesions	Postural instability	^122^
**Functional connectivity**	**BOLD fMRI**	↓↓↓ in RRMS lesions ↑↑↑ in RRMS peri-lesional area		^133^

ADC, apparent diffusion coefficient; BOLD, blood oxygenation level-dependent; Cr, creatinine; DTI, diffusion tensor imaging; EDSS, expanded disability status scale; FA, fractional anisotropy; fMRI, functional MRI; FWHM, full-width half-maximum; Glx, glutamate and glutamine; MD, mean diffusivity; MRI, magnetic resonance imaging; MRS, magnetic resonance spectroscopy; MS, multiple sclerosis; MT, magnetization transfer; MTR, magnetization transfer ratio; MTSat, quantitative MT saturation; NAA, *N*-acetyl-aspartate; NAWM, normal-appearing white matter; NODDI, neurite orientation dispersion and density imaging; ODI, orientation dispersion index; P0xy, zero displacement probability; PPMS, primary progressive MS; QSI, q-space imaging; RD, radial diffusivity; RIS, radiologically isolated syndrome; RRMS, relapsing–remitting MS; SMT, spherical mean technique; V_ax_, axonal volume fraction; v_in_, intra-neurite volume fraction.

### Diffusion based techniques

DTI provides quantitative measures of microstructural abnormalities, which have been found to be abnormal in MS when compared with healthy controls. Recent studies have reported increased magnitude of diffusion in the direction perpendicular to the main direction of fibre bundles [i.e. radial diffusivity (RD)], and reduced diffusion anisotropy [i.e. fractional anisotropy (FA)] in RRMS with acute spinal cord involvement, when compared with healthy controls, and in SPMS, when compared with clinically stable RRMS,^27,117^ suggesting reduced myelin and axonal integrity and impaired neuronal fibre coherence.^134^ A combination of DTI indices could explain up to 77% of the EDSS variability, suggesting a strong contribution of spinal cord microstructural changes to irreversible disability (Table 1).^27^ A recent study, which investigated the reproducibility of DTI-derived measures at C1–C6 between different sites, has shown the feasibility of multicentre spinal cord DTI, with adequate matching of the sequence design across sites, in particular for different manufacturers.^135^ The main advantages of spinal cord DTI are that it is simple to acquire and easy to interpret; its main limitation is that the DTI-derived measures are based on model approximations that the biological substrate is likely to violate and have low pathological specificity.

Q-space imaging (QSI) is a model-free technique that determines the voxelwise probability density function of fibre orientation, and seems to be more sensitive than conventional DTI measures at detecting MS-related abnormalities.^136^ QSI-derived indices of perpendicular diffusivity are increased and indices of parallel diffusivity are decreased in the spinal cord of early PPMS, when compared with controls, possibly reflecting increased movement of water in the direction perpendicular to the long axis of the cord, due to the breakdown of myelin and axonal membranes, even in the absence of a significant degree of spinal cord atrophy.^122,123^ Changes in QSI-derived measures are associated with different measures of clinical disability, suggesting that they reflect pathological abnormalities that contribute to neurological deficits (Table 1).^122,123^ The main limitations of QSI include the need to acquire a large number of data points, therefore necessitating long acquisition times, limited directional resolution, and difficulty in interpreting the probability density function.

**Figure 4.**
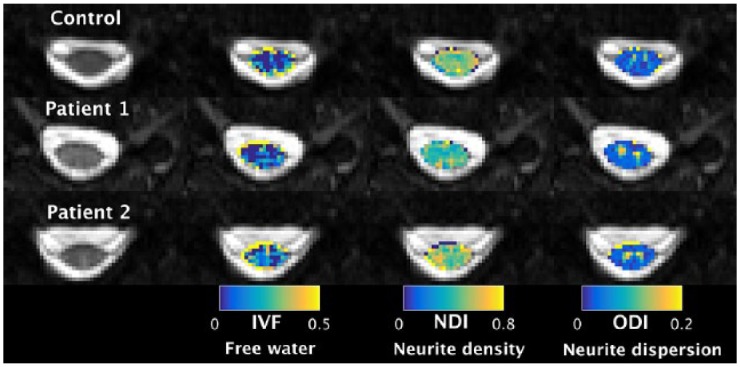
NODDI in the spinal cord. NODDI provides tissue-specific indices related to geometrical complexity of neurite architecture. Cervical spinal cord NODDI maps of IVF (estimating the amount of free water), NDI (estimating the number of neurites), and ODI (estimating the variability of neurite orientations) are shown from healthy controls and patients with MS (courtesy Dr Francesco Grussu, University College London, UK). IVF, isotropic volume fraction; MS, multiple sclerosis; NDI, neurite density index; NODDI, neurite orientation dispersion and density imaging; ODI, orientation dispersion index.

Neurite orientation dispersion and density imaging (NODDI) is a recently developed multi-compartmental diffusion model, providing microstructural indices related to geometrical complexity of neurite architecture (Figure 4).^137^ This technique applied to the spinal cord has been recently validated by comparison with histology, and a trend towards lower neurite complexity in demyelinated lesions, has been demonstrated.^138,139^ In a pilot study we found that neurite dispersion index was reduced in the spinal cord of patients with RRMS when compared with healthy controls,^124^ and a recent study has described reduced orientation index in the normal-appearing white matter and lesions of the spinal cord from six patients with MS, when compared with eight healthy controls (Table 1).^125^ The main findings of brain and spinal cord NODDI studies is that for similar value of FA there are different combinations of orientation dispersion index and neurite dispersion index, so NODDI is expected to be more pathologically specific than DTI.

Finally, an exploratory study has assessed the feasibility of the spherical mean technique (SMT), which is another multi-compartmental diffusion model, in the spinal cord in six patients with MS and eight controls (Table 1).^126^ SMT, which is feasible on standard MRI scanners, enables the mapping of the neurite density and compartment diffusivities, and is sensitive in identifying abnormal changes in MS lesions when compared with healthy white matter.^126^

An *in vivo* study of the spinal cord, which fits, studies and compares several biophysical models, similar to what has been done in the brain,^140^ would be important to establish the limitations and the advantages of each model and the clinical potential of the latest models. Reducing the acquisition times, without sacrificing the accuracy of the derived indices, may be possible with the latest techniques.^126^ The development of more advanced hardware (high-field MRI scanners), software (localization, gating, and motion compensation), and coils (such as multi-channel phased-array coils) will contribute to expand the use of diffusion-derived metrics in MS clinical practice and trials.^120^

### Techniques reflecting myelin content

MTR is a quantitative technique measuring the magnetization exchange between freely mobile protons and those associated with macromolecules such as myelin, providing an indirect estimate of myelin content, in addition to neuroaxonal integrity and water content. A large study carried out in patients with MS reported lower MTR values in patients with a higher EDSS, than those with a lower EDSS, independent of lesion load,^128^ suggesting that this measure can detect clinically relevant differences beyond conventional imaging. Reduced MTR values were found in the cervical spinal cord of 60 patients with early RRMS when compared with 34 age-matched controls, in the absence of spinal cord atrophy.^129^ This study also showed that the main contribution to low MTR levels is from the normal-appearing spinal cord tissue, since the effect of the lesions is minimal.^129^ In patients, there was a correlation between lower MTR and higher lesion load.^129^ Some evidence for reduced MTR values were also found in the cervical cord of patients with RIS,^116^ although this finding requires further confirmation. Of note, the distribution of MTR reduction in the spinal cord periphery and barycentre supports a spatial pattern of microstructural damage that resembles that in the brain,^129,141^ and suggests that MTR abnormalities in a region involving the pia mater and subpial cord occur early in the course of MS and are more marked in those with a progressive course.^142^ Clinical correlates of MTR are reported in Table 1.

Myelin water imaging has been validated as a technique that provides a marker for myelin,^143,144^ but it has been applied to the spinal cord in only a few studies. Myelin water fraction varies along the spinal cord proportionally to the white matter area fraction.^145^ In patients with cervical spondylotic myelopathy, myelin water imaging shows high specificity in detecting impaired spinal cord conduction, when compared with conventional imaging (e.g. T2 signal intensity) which only provides a measurement of the extent of spinal cord compression.^146^ In MS, spinal cord myelin water fraction decreased by 11% in PPMS, but not in healthy controls,^130^ and was associated with disability scores (Table 1),^147^ suggesting progressive demyelination in this disease subtype, that is related to progressive disability accrual. Cervical spinal cord myelin water volume fraction progressively decreases in MS, but not in NMOSD, in the absence of clinical relapses, suggesting that neurodegenerative and demyelinating processes occur continuously in MS, but not in NMOSD where inflammation might dominate.^131^

MTR and myelin water imaging appear to provide complementary information. Although MTR is not pathologically specific, it is commonly available and fast to acquire, while myelin water imaging is more specific as a myelin marker but requires more complicated post-processing and is not a sequence product.

A recent paper investigated the role of spinal cord magnetization transfer (MT) saturation (MTSat), which is a quantitative MT saturation technique, minimally affected by T1 relaxation and field inhomogeneity, and demonstrated that MTSat correlates with disability more strongly than MTR, suggesting higher sensitivity to tissue damage for future clinical applications (Table 1).^132^

Finally, quantitative MT (qMT) applied at 3T with reasonable acquisition time showed excellent grey/white contrast and sensitivity to MS pathology (lesions).^148^

### Metabolic imaging techniques

^1^H-MRS estimates the levels of metabolites, as long as they are available in relatively high concentrations.^149^ The most commonly estimated metabolites are total *N*-acetyl-aspartate (NAA; a marker of neuronal mitochondrial metabolism and, more in general, of neuronal integrity); NAA/Cr (NAA values normalized by voxel creatinine); glutamate and its precursor glutamine (Glx; whose reduction indicates chronic neuroaxonal degeneration); and *myo*-inositol (a marker of glial cell activation and proliferation). A recent investigation of MRS at 3T using *in vivo* and postmortem experiments reported an extended metabolic profile of the spinal cord, thereby indicating the rich information which can be provided by this technique.^150^

When MRS was applied to patients with early PPMS, lower NAA, lower Glx, and higher *myo*-inositol, in particular within lesions, were detected in patients compared with normal-appearing tissue in healthy controls.^122^ Metabolic changes within the spinal cord occurred in the absence of significant spinal cord atrophy, pointing towards early neuroaxonal loss and tissue remodelling, and were associated with disability measures.^122^ When including patients at different disease stages, lower NAA/Cr was associated with spinal cord atrophy and with disability progression during follow up (Table 1).^127^ Also, diffuse lesions were characterized by lower NAA/Cr when compared with focal lesions.^127^

MRS may assist with the differential diagnosis of myelopathies. It has been used to define the metabolic profile of different spinal cord tumours (e.g. strongly reduced NAA and strongly increased *myo*-inositol in the ependymoma, or absence of significant metabolic changes in extradural tumours, such as the schwannoma), and traumatic spinal cord injury (reduced NAA).^151^

^23^Na-MRS has been investigated in the brains of MS patients in several studies,^152,153^ that have shown increased total sodium concentration in the MS lesions and normal-appearing tissue in patients when compared with controls, suggesting either an expansion of the extracellular compartment as a consequence of neuroaxonal loss, or an accumulation of sodium in the swollen axonal terminals with ongoing degeneration.^114^ Advances in sodium imaging acquisition and analysis have allowed application of this metabolic technique to the spinal cord of healthy controls,^154^ and patients with MS;^155^ preliminary findings mirror brain results, with higher total sodium concentration in patients with MS than healthy controls.

### Functional MRI

Very few studies have investigated the resting state blood oxygenation level-dependent (BOLD) signal in the spinal cord mainly because its sensitivity and reliability are still suboptimal and technical limitations are significant.^156^ In a 7T fMRI study measuring the BOLD signal, spinal cord functional networks were generally intact in RRMS (Table 1). However, increased connectivity was found at the boundaries of lesions, possibly indicating compensatory changes to demyelination/axonal loss, or disruption of inhibitory spinal interneurons.^133^

## Future research

Ultra-high-field (7T) scanners have appeared in recent years. 7T MRI of the spinal cord can potentially overcome limitations of 1.5 and 3T scanners, by improving spatial resolution, increasing the contrast-to-noise ratio, and allowing better characterization of white and grey matter.^157,158^ However, 7T spinal cord MRI remains technically challenging due to motion artefacts and field inhomogeneities, and requires time-consuming acquisition and complex post-processing. New coils that reduce field inhomogeneities and allow whole spine coverage will help to overcome these limitations and develop this exciting tool in MS. Preliminary reports have shown increased sensitivity and spatial accuracy in characterizing pathology in the spinal cord than lower field MRI.^159^

In addition to spinal cord imaging at 7T, future research will focus on: (1) Developing and optimizing methods and techniques that can overcome the technical challenges posed by imaging the spinal cord, (2) Clarifying the use of asymptomatic lesions for monitoring MS and their added value to brain asymptomatic lesions, (3) Developing and optimizing quantitative MRI techniques, which provide biomarkers reflecting pathological abnormalities that contribute to disability, (4) Optimizing registration-based techniques for computing spinal cord atrophy, which will increase precision and reduce variability of spinal cord atrophy quantification and translate it into the design of clinical trials.

The ultimate goal of future spinal cord imaging research is to characterize *in vivo* axonal pathology and other pathological abnormalities in a clinical setting, thereby improving our understanding of the disease mechanisms and monitor the clinical course of MS and its response to treatment.
